# A Novel Linear Spectrum Frequency Feature Extraction Technique for Warship Radio Noise Based on Complete Ensemble Empirical Mode Decomposition with Adaptive Noise, Duffing Chaotic Oscillator, and Weighted-Permutation Entropy

**DOI:** 10.3390/e21050507

**Published:** 2019-05-18

**Authors:** Yuxing Li, Long Wang, Xueping Li, Xiaohui Yang

**Affiliations:** 1 Faculty of Information Technology and Equipment Engineering, Xi’an University of Technology, Xi’an 710048, Shaanxi, China; 2 School of Marine Science and Technology, Northwestern Polytechnical University, Xi’an 710072, Shaanxi, China; 3 School of Art and Design, Inner Mongolia University of Science & Technology, Baotou 014010, Inner Mongolia, China

**Keywords:** underwater acoustic signal, linear spectrum, frequency feature extraction, empirical mode decomposition (EMD), complete EEMD with adaptive noise (CEEMDAN), duffing chaotic oscillator (DCO), weighted-permutation entropy (W-PE), warship radio noise

## Abstract

Warships play an important role in the modern sea battlefield. Research on the line spectrum features of warship radio noise signals is helpful to realize the classification and recognition of different types of warships, and provides critical information for sea battlefield. In this paper, we proposed a novel linear spectrum frequency feature extraction technique for warship radio noise based on complete ensemble empirical mode decomposition with adaptive noise (CEEMDAN), duffing chaotic oscillator (DCO), and weighted-permutation entropy (W-PE). The proposed linear spectrum frequency feature extraction technique, named CEEMDAN-DCO-W-PE has the following advantages in comparison with other linear spectrum frequency feature extraction techniques; (i) as an adaptive data-driven algorithm, CEEMDAN has more accurate and more reliable decomposition performance than empirical mode decomposition (EMD) and ensemble EMD (EEMD), and there is no need for presetting parameters, such as decomposition level and basis function; (ii) DCO can detect the linear spectrum of narrow band periodical warship signals by way of utilizing its properties of sensitivity for weak periodical signals and the immunity for noise; and (iii) W-PE is used in underwater acoustic signal feature extraction for the first time, and compared with traditional permutation entropy (PE), W-PE increases amplitude information to some extent. Firstly, warship radio noise signals are decomposed into some intrinsic mode functions (IMFs) from high frequency to low frequency by CEEMDAN. Then, DCO is used to detect linear spectrum of low-frequency IMFs. Finally, we can determine the linear spectrum frequency of low-frequency IMFs using W-PE. The experimental results show that the proposed technique can accurately extract the line spectrum frequency of the simulation signals, and has a higher classification and recognition rate than the traditional techniques for real warship radio noise signals.

## 1. Introduction

It is very important to find underwater targets as early as possible and extract their effective features for recognition, so as to take better defensive measures and countermeasures to reduce the threat of underwater targets, such as warships and submarines [1,2,3]. There are two main components of underwater target radiated noise: the continuous spectrum of broadband noise and the discrete line spectrum. Line spectrum has higher energy level and stability than continuous spectrum. Its energy is usually concentrated in the low-frequency band. Line spectrums contain abundant underwater target parameters and motion information, which are the main basis for underwater target tracking and recognition. Line spectrum feature extraction is one of the key techniques for detecting underwater targets, especially for submarines and warships. Therefore, how to extract line spectrum features from underwater target radiated noise is always a difficult problem in complicated marine environment [4,5,6].

For a long time, classical Fourier analysis and wavelet transform have been used as the basis of underwater acoustic signal processing [7]. The characteristic parameters of underwater acoustic target signals are extracted by correlation analysis, spectral analysis and time-frequency analysis. However, these signal processing methods are not suitable for analyzing the nonstationary underwater acoustic signals, the feature extraction results cannot reflect the real features for the target signal well [8,9]. As the rapid development of signal processing technology, some signal processing methods for nonlinear and nonstationary signals are proposed, such as empirical mode decomposition (EMD) [10,11], local mean decomposition (LMD) [12,13], variational mode decomposition (VMD) [14,15], and their improved algorithms [16,17,18,19]. Some of these mode decomposition algorithms have been applied to feature extraction of underwater acoustic target signals, which can be divided into three groups based on the extracted feature information: energy feature extraction, complexity feature extraction, and frequency feature extraction.

In terms of energy feature extraction, a new energy feature extraction technique was proposed for ship-radiated noise based on ensemble EMD (EEMD) and energy distribution by Yang Hong et al. [20], which extracted energy difference between high-frequency band and low-frequency band. On the basis of reference [20], an improved energy feature extraction technique was put forward for ship-radiated noise by Li Yuxing et al. [21], which uses complete EEMD with adaptive noise (CEEMDAN) instead of EEMD and combines energy difference and energy entropy as a new hybrid energy feature. Therefore, this hybrid energy feature extraction technique has better performance of classification and recognition.

In terms of complexity feature extraction, EMD and permutation entropy (PE) were first used in feature extraction of underwater acoustic signals in 2016 [22]. On the basis of reference [22], an improved complexity feature extraction technique was put forward for underwater acoustic signals in 2017 [23], which uses VMD and multiscale PE (M-PE) instead of EMD and PE, and has the following advantages; (i) VMD can suppress mode mixing in EMD and (ii) M-PE can better reflect the difference of intrinsic mode functions (IMFs) complexity from different scales. In addition, some techniques have been proposed based on mode decomposition and complexity in the last two years [24,25].

Frequency feature extraction techniques can be divided into two categories: statistical frequency and line spectrum frequency. Two statistical frequency feature extraction techniques were put forward for ship-radiated noise, which extracted the central frequency features of maximum energy IMF by using EEMD and VMD [26,27]. In [28], a line spectrum frequency feature extraction technique for underwater acoustic signals was proposed by using duffing chaotic oscillator (DCO) and Hilbert transform, which has better performance of feature extraction in low signal noise ratio ocean environment. Combining the advantages of the above frequency feature extraction techniques, an improved line spectrum frequency feature extraction technique was put forward for underwater acoustic signals by using VMD, DCO, and a kind of PE (KPE) in 2019 [29], which can accurately extract line spectrum frequency features of low-frequency IMFs. However, this frequency feature extraction technique still has some limitations: (i) the decomposition result of VMD is affected by parameter setting and (ii) KPE cannot reflect the amplitude information of time series, which is affects the accuracy of line spectrum frequency.

For resolving these problems, this paper introduces a novel linear spectrum frequency feature extraction technique for warship radio noise based on CEEMDAN, DCO and weighted-permutation entropy (W-PE), named CEEMDAN-DCO-W-PF. The proposed technique not only retains the advantages of existing techniques, but also overcomes the disadvantages by using CEEMDAN and W-PE instead of VMD and KPE, respectively.

This paper is organized as follows. Section 2 is the introduction of basic theories, such as CEEMDAN, DCO, and W-PE. Section 3 introduces the CEEMDAN-DCO-W-PF technique; simulation and real warship radio noise data are processed by CEEMDAN-DCO-W-PF and other frequency feature extraction techniques in Section 4 and Section 5. Conclusions are drawn in Section 6.

## 2. Methods

### 2.1. CEEMDAN

CEEMDAN can provide IMFs for feature extraction of underwater acoustic signals, which attenuates the effect of mode mixing on decomposition results and eliminates the selection of parameters. We set the original underwater acoustic signal as y(t), $w_{i}(t)$ is white Gaussian noise with different amplitudes, and $E_{i}(\cdot )$ represents the i-th IMF by EMD. Then the CEEMDAN algorithm steps are as follows.

(1) New underwater acoustic signals are constructed as follows
  (1) $$y_{i}(t)=y(t)+w_{i}(t),\begin{array}{cc} & i=1,2,\cdots ,N \end{array}$$
(2) Each new underwater acoustic signal is decomposed into a first IMF and a residual item as follows
  (2) $$\left[\begin{array}{c} y_{1}(t)\\ y_{2}(t)\\ \cdots \\ y_{i}(t)\\ \cdots \\ y_{N}(t) \end{array}\right]\overset{\mathrm{Partial}\;\mathrm{EMD}}{\to }\left[\begin{array}{cc} c_{1}(t) & r_{1}(t)\\ c_{2}(t) & r_{2}(t)\\ \cdots & \cdots \\ c_{i}(t) & r_{i}(t)\\ \cdots & \cdots \\ c_{N}(t) & r_{N}(t) \end{array}\right]$$
  where $c_{i}(t)$ and $r_{i}(t)$ are the first IMF and residual item of $y_{i}(t)$ by EMD.
(3) The first IMF of y(t) can be expressed as follows
  (3) $${\tilde{c}}_{1}(t)=\frac{1}{N}\sum_{i=1}^{N}c_{i}(t)$$
(4) The residual item of ${\tilde{c}}_{1}(t)$ can be expressed as
  (4) $${\tilde{r}}_{1}(t)=y(t)-{\tilde{c}}_{1}(t)$$
(5) Each $w_{i}(t)$ can be decomposed as follows
  (5) $$\left[\begin{array}{c} w_{1}(t)\\ w_{2}(t)\\ \cdots \\ w_{i}(t)\\ \cdots \\ w_{N}(t) \end{array}\right]\overset{\mathrm{EMD}}{\to }\left[\begin{array}{ccccc} c_{1}^{w_{1}}(t) & c_{2}^{w_{1}}(t) & \cdots & c_{j}^{w_{1}}(t) & r^{w_{1}}(t)\\ c_{1}^{w_{2}}(t) & c_{2}^{w_{2}}(t) & \cdots & c_{j}^{w_{2}}(t) & r^{w_{2}}(t)\\ \cdots & \cdots & \cdots & \cdots & \cdots \\ c_{1}^{w_{i}}(t) & c_{2}^{w_{i}}(t) & \cdots & c_{j}^{w_{i}}(t) & r^{w_{i}}(t)\\ \cdots & \cdots & \cdots & \cdots & \cdots \\ c_{1}^{w_{N}}(t) & c_{2}^{w_{N}}(t) & \cdots & c_{j}^{w_{N}}(t) & r^{w_{N}}(t) \end{array}\right]$$
(6) $E_{1}(w_{i}(t))$ can be expressed as
  (6) $$E_{1}(w_{i}(t))={\left(\begin{array}{cccccc} c_{1}^{w_{1}}(t) & c_{1}^{w_{2}}(t) & \cdots & c_{1}^{w_{i}}(t) & \cdots & c_{1}^{w_{N}}(t) \end{array}\right)}^{T}$$
(7) Construct $y_{i}^{1}(t)$ as follows
  (7) $$y_{i}^{1}(t)={\tilde{r}}_{1}(t)+E_{1}(w_{i}(t))$$
(8) Decompose $y_{i}^{1}(t)$ to obtain the first IMF as follows
  (8) $$y_{i}^{1}(t)={\tilde{r}}_{1}(t)+\left[\begin{array}{c} c_{1}^{w_{1}}(t)\\ c_{1}^{w_{2}}(t)\\ \cdots \\ c_{1}^{w_{i}}(t)\\ \cdots \\ c_{1}^{w_{N}}(t) \end{array}\right]\overset{\mathrm{Partial}\;\mathrm{EMD}}{\to }\left[\begin{array}{c} c_{1}^{y_{1}}(t)\\ c_{1}^{y_{2}}(t)\\ \cdots \\ c_{1}^{y_{i}}(t)\\ \cdots \\ c_{1}^{y_{N}}(t) \end{array}\right]$$
(9) The second IMF of y(t) can be expressed as follows
  (9) $${\tilde{c}}_{2}(t)=\frac{1}{N}\sum_{i=1}^{N}c_{1}^{y_{i}}(t)$$
(10) The residual item of ${\tilde{c}}_{2}(t)$ can be expressed as
  (10) $${\tilde{r}}_{2}(t)={\tilde{r}}_{1}(t)-{\tilde{c}}_{2}(t)$$
(11) Calculate the other IMFs according to the following formulas
  (11) $$y_{i}^{j}(t)={\tilde{r}}_{j}(t)+E_{j}(w_{i}(t))$$
  (12) $${\tilde{c}}_{j+1}(t)=\frac{1}{N}\sum_{i=1}^{N}c_{j}^{y_{i}}(t)$$
  (13) $${\tilde{r}}_{j+1}(t)={\tilde{r}}_{j}(t)-{\tilde{c}}_{j+1}(t)$$
(12) The original underwater acoustic signal y(t) can be expressed as
  (14) $$y(t)=\sum_{j=1}^{L}{\tilde{c}}_{j}(t)+r(t)$$
  where L and r(t) represent the number of ${\tilde{c}}_{j}(t)$ and the residual item of y(t).

### 2.2. DCO

The DCO system model was originally derived from a nonlinear dynamic equation describing the forced oscillation of a damped simple pendulum. The nonlinear dynamic equation is as follows

(15) $$ml\theta^{\prime \prime }+rl\theta^{\prime }+mg\sin\theta =f\cos\omega t$$

where m is mass and l and θ represent the length and the swing angle of the simple pendulum, respectively. Divide formula (15) by mg and set $\omega_{0}^{2}$ equal to $\frac{g}{l}$; the renewal equation is as follows

(16) $$\frac{1}{\omega_{0}^{2}}\theta^{\prime \prime }+\frac{r}{m\omega_{0}^{2}}\theta^{\prime }+\sin\theta =\frac{f}{mg}\cos\omega t$$

Set $\frac{\omega }{\omega_{0}}$, $\omega_{0}t$, $\frac{r}{m\omega_{0}}$, and $\frac{f}{mg}$ equal to Ω, T, 2β, and F; the renewal equation is as follows

(17) $$\frac{d\theta^{2}}{dT^{2}}+2\beta \frac{d\theta }{dT}+\sin\theta =F\cos\Omega T$$

sinθ is replaced according to Maclanrin polynomial expansion as follows

(18) $$a_{1}x+a_{3}x^{3}+\cdots +a_{2p+1}x^{2p+1}\;(a_{1}=-1,a_{2}=1,a_{5}=\cdots =a_{2p+1}=0)$$

Set 2β, θ, and F equal to k, x, and r, the DCO equation is as follows

(19) $$x^{\prime \prime }+kx^{\prime }-x+x^{3}=r\cos\omega t$$

where k and $-x+x^{3}$ are the damping ratio and the nonlinear resilience item, respectively, and r and ω are the amplitude and the angular frequency of the driving force, respectively.

DCO has abundant nonlinear dynamic characteristics because of $-x+x^{3}$. As r increases from 0, the system has four stages: the homoclinic orbit stage, the bifurcation stage, the chaos stage, and the great periodic stage. When r is greater than the threshold value $r_{d}$, the system enters into a great periodic stage from a chaos stage. The threshold value $r_{d}$ from DCO is different according to the angular frequency of the driving force ω [28]. The steps for detecting the line spectrum frequency of the periodic signal based DCO are as follows [29]:

(1) Mixed signal is added to the system, which is made up of periodic signal s and noise signal n. The renewal equation is as follows
  (20) $$x^{\prime \prime }+kx^{\prime }-x+x^{3}=r\cos\omega t+s+n$$
(2) Initialize x(0) and $x^{\prime }(0)$ to 0 and set k equal to 0.5. Then, we use the fourth-order Runge–Kutta method to solve the above equation.
(3) The line spectrum frequency of the periodic signal depends on the stage of the system. If the system is in the great periodic stage, the line spectrum frequency of the periodic signal is close to ω.

DCO can help us to determine the range of line spectrum frequency. However, the final line spectrum frequency depends on the value of W-PE.

### 2.3. W-PE

W-PE was proposed by Fadlallah et al. in 2013 [30]. Like PE, W-PE is also a nonlinear dynamic parameter based on complexity measure. However, they have some similarities and differences as follows.

**Figure 1:**
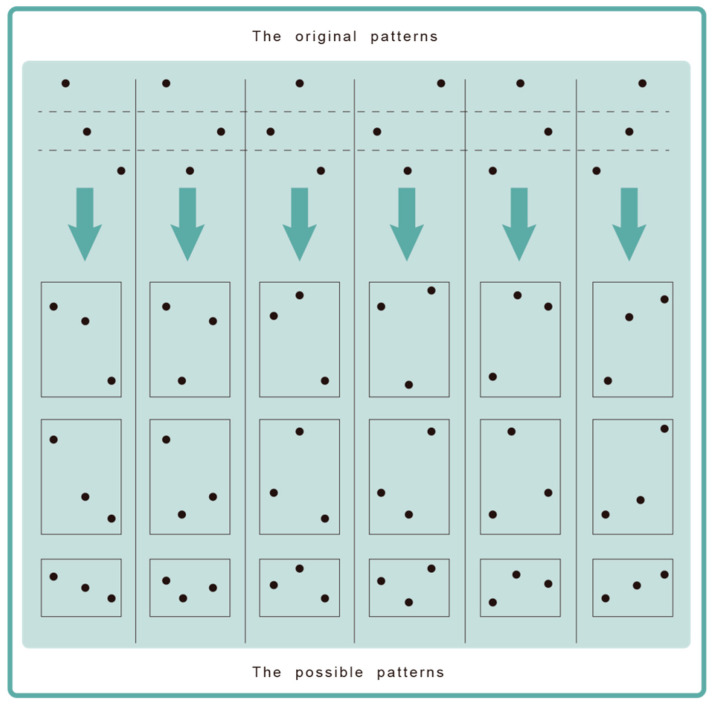
The original patterns for permutation entropy (PE) and the possible patterns for weighted-permutation entropy (W-PE).

(1) Both PE and W-PE include four steps: phase space reconstruction, ascending order, entropy calculation, and normalization. Except for the entropy calculation, the other three steps between PE and W-PE are exactly the same.
(2) Compared with the original patterns for PE, W-PE has more possible patterns because of the introduction of amplitude information. For example, when the embedding dimension is 3, the original patterns for PE and the possible patterns for W-PE are shown in Figure 1.
(3) Entropy calculations are different. The equations of PE and W-PE are as follows
  (21) $$\left\{ \begin{array}{l} H_{PE}=-\sum_{i=1}^{m!}P(\pi_{i})\ln P(\pi_{i})\\ H_{W-PE}=-\sum_{i=1}^{m!}P_{\omega }(\pi_{i})\ln P_{\omega }(\pi_{i}) \end{array}\right.$$
  where m is the embedded dimension and $P(\pi_{i})$ and $P_{\omega }(\pi_{i})$ represent the i-th probability of PE and W-PE, respectively, as follows
  (22) $$\left\{ \begin{array}{l} P(\pi_{i})=\frac{f(\pi_{i})}{\sum_{i=1}^{m!}f(\pi_{i})}\\ P_{\omega }(\pi_{i})=\frac{f_{\omega }(\pi_{i})}{\sum_{i=1}^{m!}f_{\omega }(\pi_{i})} \end{array}\right.$$
  where $f(\pi_{i})$ and $f_{\omega }(\pi_{i})$ are the frequency for the i-th permutation of PE and W-PE, respectively, $f(\pi_{i})$ can be obtained directly by statistics, and $f_{\omega }(\pi_{i})$ can be expressed as
  (23) $$f_{\omega }(\pi_{i})=\sum_{s}^{S}f(\pi_{i}(s))\omega_{i}(s)\;(s=0,1,\cdots ,S)$$
  where S and $\omega_{i}(s)$ are the number of possible patterns for the i-th permutation and corresponding weight values, respectively. $\omega_{i}(s)$ can be obtained by calculating the variance of the vector $X_{j}$ as follows
  (24) $$\left\{ \begin{array}{l} \omega_{i}(\pi_{s})=\frac{1}{m}\sum_{k=1}^{m}{\left(x_{\left(j+(k-1)\tau \right)}-{\overset{¯}{X}}_{j}\right)}_{}^{2}\\ {\overset{¯}{X}}_{j}=\frac{1}{m}\sum_{k=1}^{m}x_{\left(j+(k-1)\tau \right)} \end{array}\right.$$
  where ${\overset{¯}{X}}_{j}$ is the mean of the vector $X_{j}$.

## 3. Linear Spectrum Frequency Feature Extraction Technique for Warship Radio Noise

**Figure 2:**
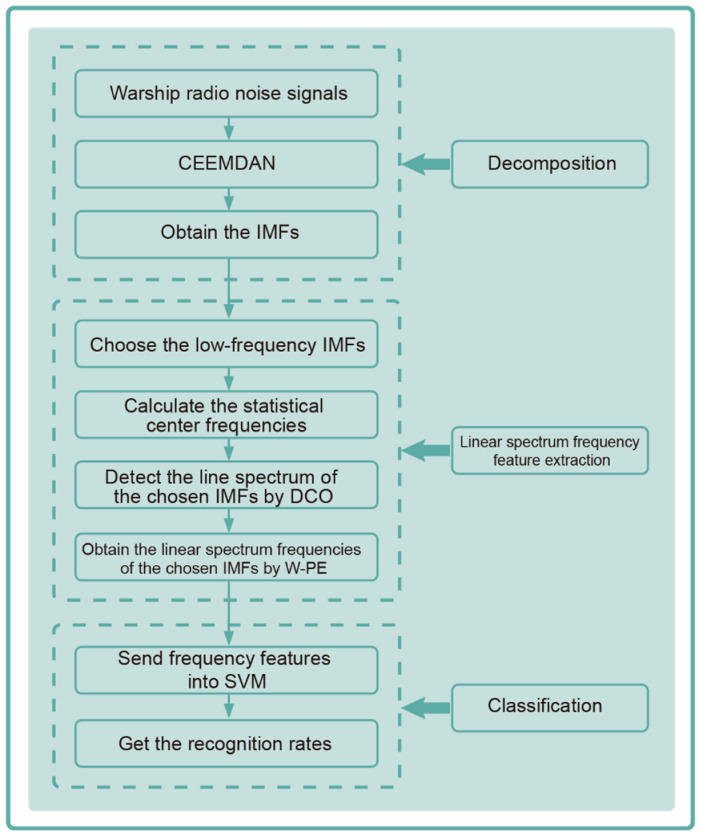
The flow chart of CEEMDAN-DCO-W-PE.

According to the theoretical analysis of CEEMDAN, DCO, and W-PE, a novel linear spectrum frequency feature extraction technique for warship radio noise is presented, named CEEMDAN-DCO-W-PE. The flow chart of CEEMDAN-DCO-W-PE is shown in Figure 2. The specific steps of the linear spectrum frequency feature extraction technique are as follows:

Stage 1: Decomposition.

(1) Different types of warship radio noise signals are measured through hydrophones.
(2) Warship radio noise signals are decomposed by CEEMDAN, then we can obtain the IMFs from high frequency to low frequency.

Stage 2: Linear spectrum frequency feature extraction.

(1) Linear spectrum frequency of warship radio noise is usually in the low-frequency band, so we choose the low-frequency IMFs for further study.
(2) We calculate the statistical center frequencies of the chosen IMFs as the initial detection frequencies of DCO.
(3) Detect the line spectrum of the chosen IMFs by DCO, we can obtain the range of line spectrum frequency.
(4) Obtain the linear spectrum frequencies of the chosen IMFs by W-PE. We regard the frequency corresponding to the minimum value of W-PE for DCO output as the line spectrum frequency.

Stage 3: Classification.

(1) Send frequency features into support vector machine (SVM).
(2) Acquire the recognition rates of different types of warship radio noise signals by training samples and testing samples.

## 4. Linear Spectrum Frequency Feature Extraction of Simulation Signals

### 4.1. CEEMDAN of Simulation Signals

**Figure 3:**
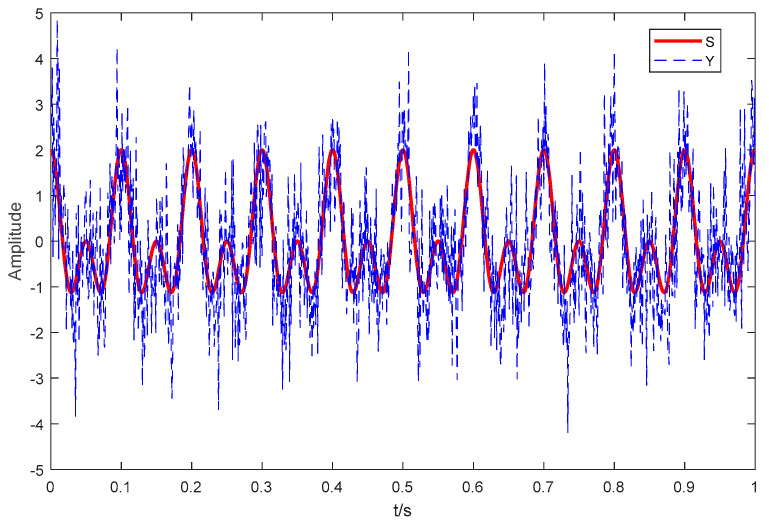
The time domain waveforms of S and Y.

**Figure 4:**
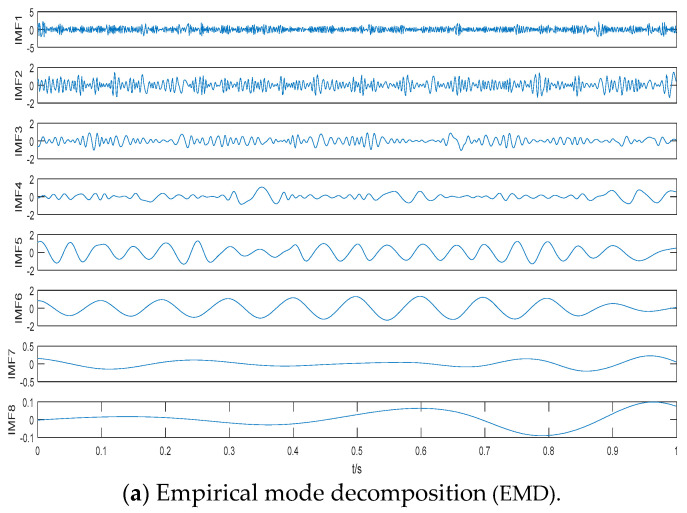

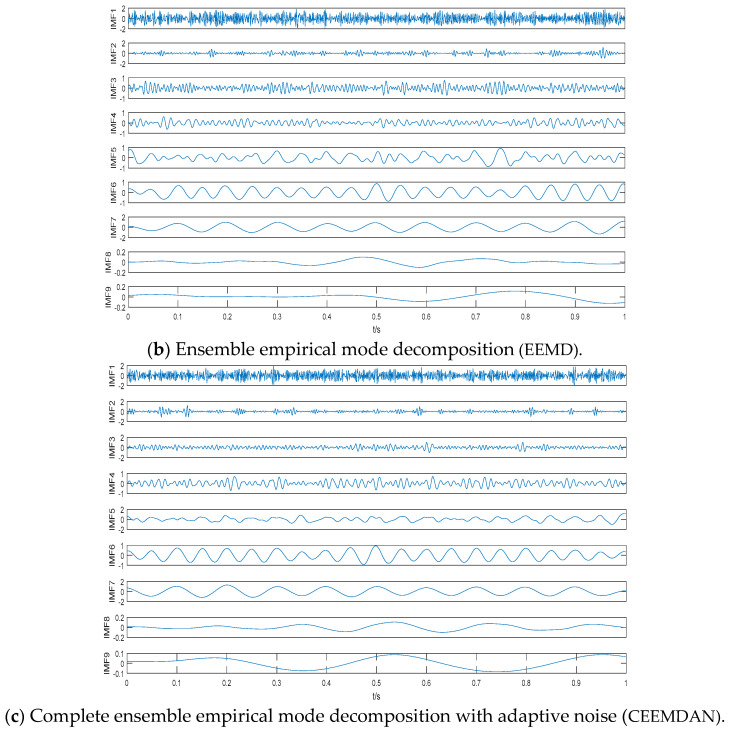
Decomposition results of different algorithms.

We apply EMD, EEMD, and CEEMDAN to simulation signals. The simulation signals are as follows

(25) $$\left\{ \begin{array}{l} S=\cos(20\pi t)+\cos(40\pi t)\\ Y=S+randn(t) \end{array}\right.$$

where the signal S consists of two cosine signals with frequencies of 10 Hz and 20 Hz, and signal Y, with sampling frequency of 1 KHz, consists of signal S and standard Gaussian white noise randn(t). The time domain waveforms of S and Y with 0 dB are shown in Figure 3. As seen in Figure 3, the signal S is flooded in the standard Gaussian white noise. Decomposition results of different algorithms are shown in Figure 4. As seen in Figure 4, we obtain eight IMFs by EMD and nine IMFs by EEMD and CEEMDAN. Table 1 is the statistical center frequency distribution of IMFs by EMD, EEMD, and CEEMDAN. The statistical center frequency is defined in [26]. As seen in Table 1 and Figure 4, the cosine signal with frequency of 10 Hz corresponds to IMF6 by EMD and IMF7 by EEMD and CEEMDAN, and the cosine signal with frequency of 20 Hz corresponds to IMF5 by EMD and IMF6 by EEMD and CEEMDAN. Comparing the statistical center frequencies of different algorithms, CEEMDAN can more accurately reflect the line spectrum frequencies of the simulated signal S than EMD and EEMD.

### 4.2. Linear Spectrum Frequency Feature Extraction of the Cosine Signal with Frequency of 10 Hz

**Figure 5:**
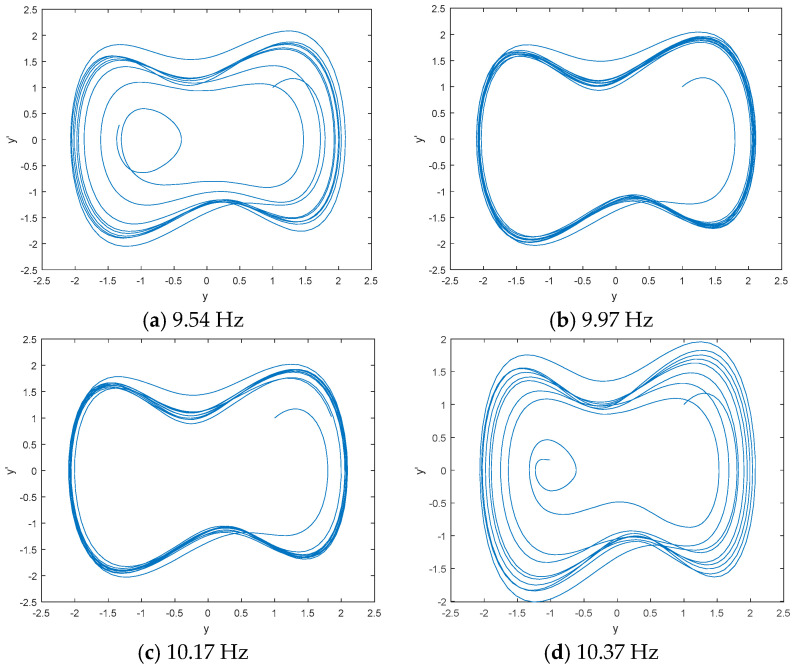
The phase space tracks of IMF7 under different driving force frequencies.

We use DCO to detect the line spectrum of IMFs from low-frequency IMF to high-frequency IMF based on the statistical center frequency of IMFs by CEEMDAN. The results show that there is no line spectrum in IMF8 and IMF9; the first line spectrum is in IMF7. Figure 5 is the phase space tracks of IMF7 under different driving force frequencies. As seen in Figure 5, the phase space tracks are under great periodic stage with the driving force frequencies of 9.97 Hz and 10.17 Hz. We calculate the PEs and W-PEs of the DCO outputs under great periodic stage with different driving force frequencies. Table 2 is the complexity distribution of IMF7 under different driving force frequencies. As seen in Table 2, the minimum values of PE and W-PE correspond to the frequency of 9.95 Hz and 9.98 Hz, which can reflect the real line spectrum frequency of IMF7.

### 4.3. Linear Spectrum Frequency Feature Extraction of the Cosine Signal with Frequency of 20 Hz

**Figure 6:**
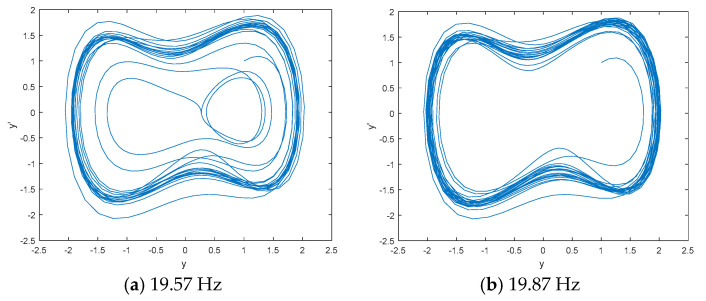

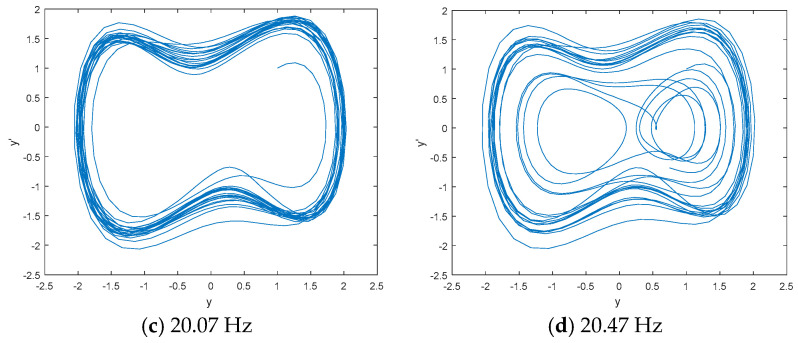
The phase space tracks of IMF6 under different driving force frequencies.

DCO is used to detect the line spectrum of IMF6 by CEEMDAN based on the statistical center frequency of 19.87 Hz. Figure 6 shows the phase space tracks of IMF6 under different driving force frequencies. As seen in Figure 6, the phase space tracks are under great periodic stage with the driving force frequencies of 19.87 Hz and 20.07 Hz. We calculate the PEs and W-PEs of the DCO outputs under great periodic stage with different driving force frequencies. Table 3 is the complexity distribution of IMF6 under different driving force frequencies. As seen in Table 3, the minimum values of PE and W-PE correspond to frequencies of 19.95 Hz and 19.97 Hz, which are close to the real line spectrum frequency of 20 Hz.

### 4.4. Comparison of Frequency Feature Extraction Techniques

We compared different frequency feature extraction techniques to prove the effectiveness of CEEMDAN-DCO-W-PE. We name the frequency feature extraction techniques using statistical center frequency and three decomposition algorithms as EMD-TCF, EEMD-TCF, and CEEMDAN-TCF. The linear spectrum frequency feature extraction technique based on CEEMDAN, DCO, and PE is named CEEMDAN-DCO-PE. The frequency feature extraction results of different techniques are listed in Table 4. As seen in Table 4, two line spectrum feature extraction techniques based on CEMDAN and DCO are superior to EMD-TCF, EEMD-TCF, and CEEMDAN-TCF; the line spectrum frequencies obtained by CEEMDAN-DCO-W-PE are the most accurate results.

## 5. Linear Spectrum Frequency Feature Extraction of Warship Radio Noise Signals

### 5.1. CEEMDAN of Warship Radio Noise Signals

**Figure 7:**
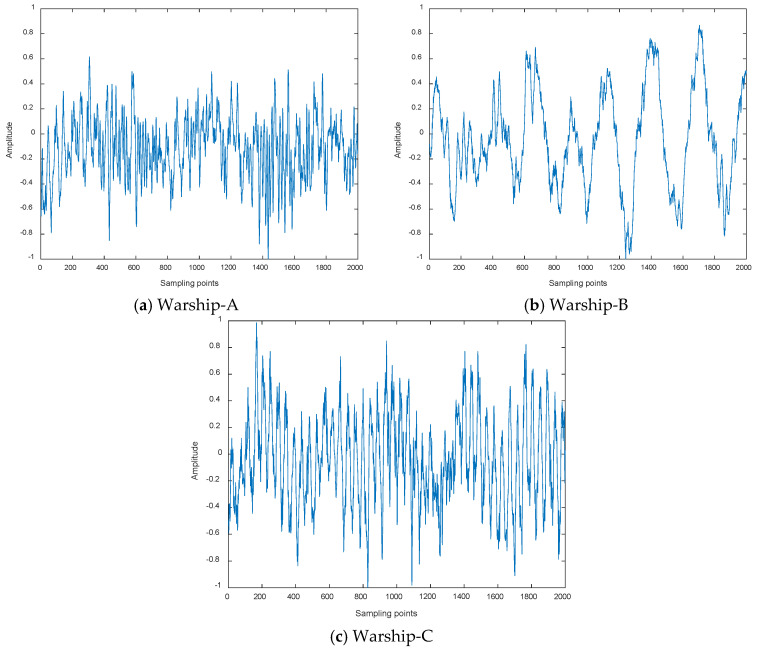
Time-domain waveforms for warships.

**Figure 8:**
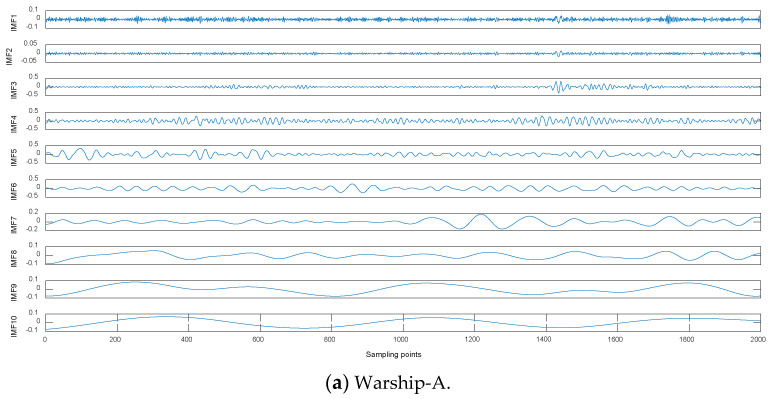

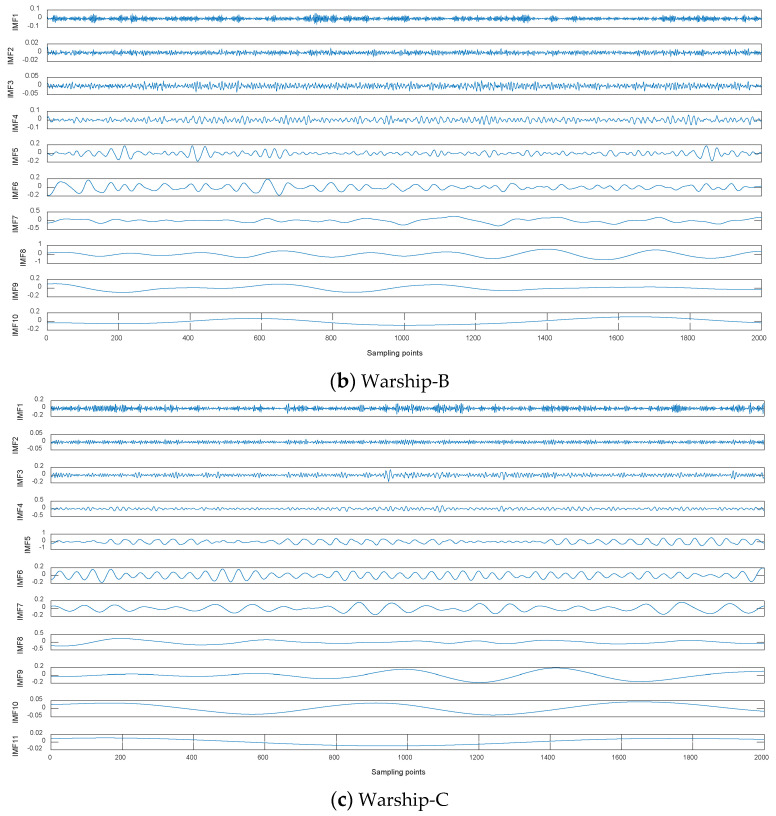
Decomposition results for warships.

Warship radio noise source is divided into three categories: mechanical noise, propeller noise and hydrodynamic noise. Warship radio noise signals contain abundant line spectrum components, which can reflect their real physical characteristics. CEEMDAN-DCO-W-PE is carried out on warship-A, warship-B, and warship-C. We measured warship radio noise signals at level 1 sea state by hydrophones. When one of the warships is running, the other warships remain out of work. Time-domain waveforms and decomposition results by CEEMDAN for warships are shown in Figure 7 and Figure 8, respectively. As seen in Figure 7 and Figure 8, the number of sampling points is 2000 and the numbers of IMFs for warships by CEEMDAN are 10, 10, and 11. For the sake of convenience we choose the low-frequency IMF10 for the research. The frequency feature extraction results of IMF10 for warships by CEEMDAN-TCF are shown in Table 5. As seen in Table 5, the statistical center frequencies of three warship signals are different to some extent.

### 5.2. Linear Spectrum Frequency Feature Extraction of IMF10

**Figure 9:**
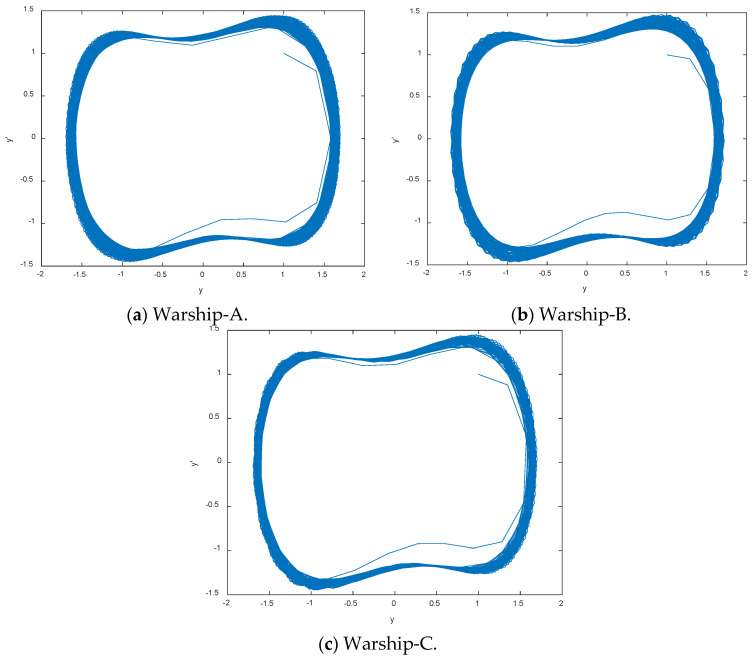
The great periodic stages of IMF10 for warships.

DCO and W-PE are used to detect and determine the line spectrum of IMF10 for warships based on the statistical center frequencies in Table 5. When the phase space track and W-PE of DCO output are in great periodic stage and the minimum value, we can obtain the great periodic stages of and the frequency feature extraction results of IMF10 for warships by CEEMDAN-DCO-W-PE in Figure 9 and Table 6. As seen in Table 6, the frequency feature extraction results of the same warship signals by CEEMDAN-DCO-W-PE are different from the ones by CEEMDAN-TCF in Table 5.

### 5.3. Comparison of Frequency Feature Extraction Techniques

**Figure 10:**
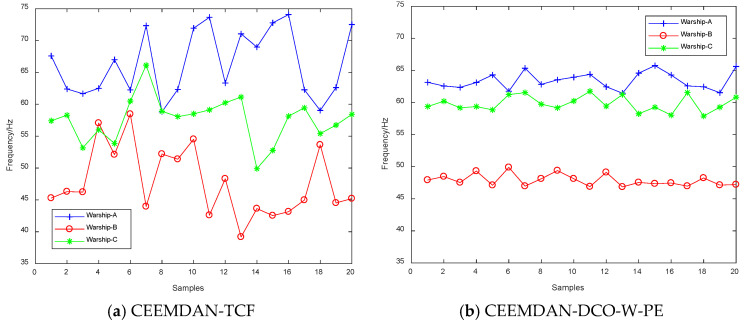
The frequency feature distributions of CEEMDAN-TCF and CEEMDAN-DCO-W-PE.

**Figure 11:**
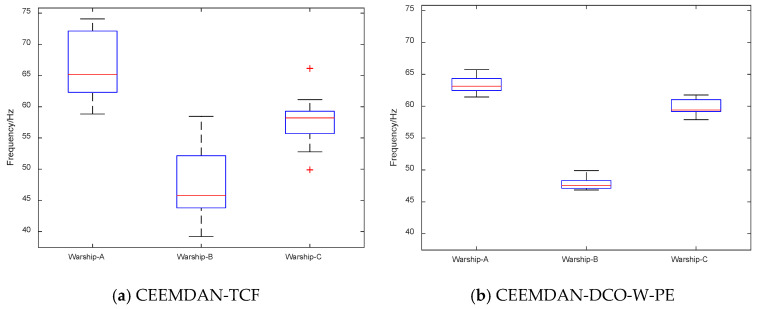
The frequency feature boxplots of CEEMDAN-TCF and CEEMDAN-DCO-W-PE.

First, we extract the frequency features of 20 samples for each warship by CEEMDAN-TCF and CEEMDAN-DCO-W-PE. The frequency feature distributions and boxplots of CEEMDAN-TCF and CEEMDAN-DCO-W-PE are shown in Figure 10 and Figure 11. As seen in Figure 10 and Figure 11, for the same warship signals, the frequency features by CEEMDAN-TCF have a larger fluctuation range than the ones by CEEMDAN-DCO-W-PE.

We increase the number of samples to 100 for each warship, and added a comparison with EMD-TCF, EEMD-TCF and CEEMDAN-DCO-PE. SVM with polynomial kernel function was used for the classification of three kinds of warships. The number of training samples and test samples are 50 and 50 for each warship. Finally, we can get the classification results by five frequency feature extraction techniques in Table 7. As seen in Table 7, the frequency feature extraction techniques based on CEEMDAN are better than ones based on EMD and EEMD, which have the recognition rates of more than 80%; two line spectrum feature extraction techniques based on CEEMDAN and DCO is superior to EMD-TCF, EEMD-TCF, and CEEMDAN-TCF, which have the recognition rates of more than 90%; the proposed CEEMDAN-DCO-W-PE has the highest classification accuracy.

## 6. Conclusions

A novel linear spectrum frequency feature extraction technique for warship radio noise is proposed based on CEEMDAN, DCO and W-PE. The crucial contributions of CEEMDAN-DCO-W-PE are as follows:

(1) CEEMDAN is used to decompose warship radio noise, which is a fully adaptive algorithm without selecting parameters.
(1) Compared with traditional DCO, DCO combined with CEEMDAN can extract the IMF line spectrum frequency features based on the statistical center frequencies of IMFs, which is more conducive to distinguishing different kinds of signals.
(3) W-PE combined DCO is first used in underwater acoustic signal processing, proving better capabilities to determine the final line spectrum frequency.
(4) Compared with other frequency feature extraction techniques, CEEMDAN-DCO-W-PE has better performance for simulation signals and actual warship radio noise signals. The classification recognition rate for the three kinds of warship radio noise signal is 92.75%.

## Figures and Tables

**Table 1:** The statistical center frequency distribution of intrinsic mode functions (IMFs) by empirical mode decomposition EMD, empirical EMD (EEMD) and complete ensemble empirical mode decomposition with adaptive noise (CEEMDAN).

	IMF1	IMF2	IMF3	IMF4	IMF5	IMF6	IMF7	IMF8	IMF9
EMD	332.1 Hz	157.8 Hz	79.27 Hz	27.72 Hz	20.24 Hz	10.17 Hz	5.153 Hz	2.632 Hz	-
EEMD	327.9 Hz	179.8 Hz	126.4 Hz	73.67 Hz	26.01 Hz	20.18 Hz	10.75 Hz	4.972 Hz	3.316 Hz
CEEMDAN	337.1 Hz	191.7 Hz	125.6 Hz	74.32 Hz	25.17 Hz	19.87 Hz	9.94 Hz	5.098 Hz	3.493 Hz

**Table 2:** The complexity distribution of IMF7 under different driving force frequencies.

	9.94 Hz	9.95 Hz	9.96 Hz	9.97 Hz	9.98 Hz	9.99 Hz	10 Hz	10.01 Hz
PE	0.8038	0.8031	0.8035	0.8035	0.8041	0.8044	0.8045	0.8048
W-PE	0.6949	0.695	0.6951	0.6949	0.6948	0.695	0.6951	0.6952

**Table 3:** The complexity distribution of IMF6 under different driving force frequencies.

	19.94 Hz	19.95 Hz	19.96 Hz	19.97 Hz	19.98 Hz	19.99 Hz	20 Hz	20.01 Hz
PE	0.8882	0.8878	0.8883	0.8885	0.8887	0.8886	0.8887	0.8891
W-PE	0.7033	0.7032	0.7031	0.7028	0.7033	0.7035	0.7037	0.7041

**Table 4:** The frequency feature extraction results of different techniques.

	EMD-TCF	EEMD-TCF	CEEMDAN-TCF	CEEMDAN-DCO-PE	CEEMDAN-DCO-W-PE
IMF7	10.17 Hz	10.75 Hz	9.94 Hz	9.95 Hz	9.98 Hz
IMF6	20.24 Hz	20.18 Hz	19.87 Hz	19.95 Hz	19.97 Hz

**Table 5:** The frequency feature extraction results of IMF10 for warships by CEEMDAN-TCF.

Warship-A	Warship-B	Warship-C
67.58 Hz	45.29 Hz	57.41 Hz

**Table 6:** The frequency feature extraction results of IMF10 for warships by CEEMDAN-DCO-W-PE.

Warship-A	Warship-B	Warship-C
63.15 Hz	47.92 Hz	59.38 Hz

**Table 7:** The classification results by five frequency feature extraction techniques.

EMD-TCF	EEMD-TCF	CEEMDAN-TCF	CEEMDAN-DCO-PE	CEEMDAN-DCO-W-PE
69.5%	70.25%	82.5%	90.25%	92.75%
